# Resource allocation of in vitro fertilization: a nationwide register-based cohort study

**DOI:** 10.1186/1472-6963-7-210

**Published:** 2007-12-21

**Authors:** Reija Klemetti, Mika Gissler, Tiina Sevón, Elina Hemminki

**Affiliations:** 1 STAKES, National Research and Development Centre for Welfare and Health, Health Services Research, Helsinki, Finland; 2 STAKES, National Research and Development Centre for Welfare and Health, STAKES Information, Helsinki, Finland; 3 University of Tampere, Finland

## Abstract

**Background:**

Infertility is common and in vitro fertilization (IVF) is a widely used treatment. In IVF the need increases and the effectiveness and appropriateness decrease by age. The purpose of this study was to describe allocation of resources for IVF by women's age, socioeconomic position, area of residence and treatment sector (public vs. private) and to discuss how fairly the IVF resources are allocated in Finland.

**Methods:**

Women who received IVF between 1996 and 1998 (N = 9175) were identified from the reimbursement records of the Social Insurance Institution (SII). Information on IVF women's background characteristics came from the Central Population Register and the SII, on treatment costs from IVF clinics and the SII, and on births from the Medical Birth Register. The main outcome measures were success of IVF by number of cycles and treated women, expenditures per IVF cycles, per women, per live-birth, and per treatment sector, and private and public expenditures. Expenditures were estimated from health care visits and costs.

**Results:**

During a mean period of 1.5 years, older women (women aged 40 or older) received 1.4 times more IVF treatment cycles than younger women (women aged below 30). The success rate decreased by age: from 22 live births per 100 cycles among younger women to 6 per 100 among older women. The mean cost of a live birth increased by age: compared to younger women, costs per born live birth of older women were 3-fold. Calculated by population, public expenditure was allocated most to young women and women from the highest socioeconomic position. Regional differences were not remarkable.

**Conclusion:**

Children of older infertile women involve more expense due to the lower success rates of IVF. Socioeconomic differences suggest unfair resource allocation in Finland.

## Background

Equity in health care assumes equal access to care based on need regardless of the patients' background characteristics. In impaired fertility, a dilemma arises from the increase with age of the need for health care, while its health impact decreases. Women's ability to spontaneously become pregnant and have a live birth decreases with age, leading to a greater need for infertility treatment such as in vitro fertilization (IVF, including intracytoplasmic sperm injections (ICSI) and frozen embryo transfers), although the success in IVF also decreases by age [1]. Furthermore, the health risks for the woman and baby increase by age. Decreased fertility is common; about 10–15% of couples are estimated to experience it at some point in their lives [2]. In Finland, besides age, no other significant socioeconomic or regional differences in infertility rates have been found [3].

IVF is costly and in countries where IVF is offered only in the private sector its availability depends on a couple's ability to pay [4,5]. In France, where IVF costs are fully covered by public resources, the use of IVF did not differ according to women's socioeconomic position [6]. In countries where IVF services are offered both in the public and in the private sector, wealthy couples can shorten their waiting times by using services in both sectors [7]. In Finland, the private sector is an important provider of IVF: over 60% of all IVF treatments are provided by private clinics [8]. However, the use of private services is partly covered by national health insurance: 60% of doctors' fees and a part of the cost of the examinations are reimbursed according to a fixed scale of charges. The part of the fee that exceeds the fixed charge is not reimbursable. In the public sector, patients pay a small user charge for their clinic visits. In both sectors about 50% of the drug costs are reimbursed by the Social Insurance Institution.

A key question in regard to fairness or equity in infertility treatment is how scarce health care resources can be distributed equitably with the maximum benefit to public health [9]. How can health care resources be allocated fairly? Should women with "greater need" but less chance of success be favoured or should women with "more benefit" (younger and better chance of success) be favoured? Health care costs for IVF treatment by women's age have been described previously [10] but empirical data that would quantify this question have not been given, nor has the distribution of private and public expenditure been estimated. The purpose of this study is to describe the allocation of IVF resources by women's age, socioeconomic position, area of residence, and treatment sector (public vs. private), as well as to discuss whether the IVF resources are allocated fairly in Finland.

## Methods

The cohort of women having received IVF between 1996 and 1998 in Finland (N = 9175) has already been described [11], as well as their background characteristics [7]. In brief, using a pre-designed algorithm (presented in detail earlier, [11]), women were identified from the reimbursement files of the Social Insurance Institution (SII), which covers the whole Finnish population. According to the algorithm all women born between 1940 and 1981 having received reimbursements for any of the drugs in the following groups during the 1996–1998 period were picked from the Drug Register: clomifen, gonadotrophin, GnRH-agonist, and a progesterone drug Lugesteron. For the women having bought drugs in the above-mentioned groups, drugs from the following groups were additionally included: human chorionic gonadotrophin, eastradiol and a progestin drug (dydrogesterone). For each woman the treatment cycles were formed by using this drug data. The cycles were linked to the Procedure Register, with codes for ovum pick-up, cultivation of ovum, intracytoplasmic sperm injection, and embryo transfer. By using all the gathered information, the cycles were classified into other ART (assisted reproduction including ovulation induction with or without insemination) and IVF by using the specific inclusion criteria [11].

The socioeconomic position of the women was defined by using their own occupation as detailed in the Central Population Registry, and classified automatically into five categories according to the national classification compiled by Statistics Finland: upper white-collar workers, lower white-collar workers, blue-collar workers, others (entrepreneurs, students, pensioners, unemployed women and women with an unclassified position) and unknown position [12]. The sector (public vs. private) was defined by using information on reimbursements and prescribing physicians' codes. All treatment cycles that were started after the beginning of January 1996 and before the end of December 1998 were included. The mean follow-up time was 1.5 years (range 0.5–3 years). Health of IVF women were followed until the end of 2000 by using different health care registers [13].

Direct costs of IVF treatments (see Additional file 1: Type of IVF cycle, expenditures included in cost calculations in each type of the cycle, as well as type and data source of expenditures by the care sector) were obtained from the SII, a private clinic (one of the largest private infertility clinics, PRCL), the Helsinki University Central Hospital (HUCH) and an earlier Finnish study by Koivurova 2005 (STUK) [14]. Direct costs included medications, visits, routine examinations (radiological and laboratory tests), interventions including ovum pick-up, ovum cultivation, ICSI, and embryo transfers, as well as cost of equipment and trained staff. Expenditures were partly based on average costs (AVER) in clinics, partly on estimations (EST) and partly on exact paid and reimbursed costs (REAL, see Additional file 1). About 30% of interrupted cycles in the private sector included ovum pick-up, and this was extrapolated to the public sector, for which this data was not available. ICSI was used in 21% of cycles in the private sector and because 65% of ICSIs were made in the private sector during the study period, we estimated that in the public sector 19% of all cycles were ICSI. Indirect costs such as costs for travel and sick leave were not included.

Private expenditures include costs paid by the woman (excluding reimbursements from the SII). Public expenditures include costs paid by the health care system (excluding user charges). All costs and reimbursements have been inflated to correspond to 2005 prices (in euros) using a consumer price index compiled by Statistics Finland.

Data on women were linked to the Finnish Medical Birth Register to identify live births resulting from IVF. Data on identified children were linked to other health care registers until the end of 2004 to follow-up the health of IVF children [15]. To measure treatment success, live births per initiated cycle (all live births per all cycles) and per treated woman (only one live birth per woman) were calculated. Multiple pregnancies with two or more live infants were calculated as one. The mean price of different types of IVF cycles by care sector were calculated as well as private, public, and total expenditures by women's background characteristics and by live births. To count expenditure by population groups to show distribution of IVF expenditures across all women in the total population, we used the mean population of females aged 20–49 in Finland by socioeconomic position and area of residence according to census information for 1995 and 2000 available from Statistics Finland. Men were not included, because regardless of male or female factor infertility, in most cases the women are treated (drugs, ovum pick-ups, and embryo transfers) and expenditures of treatments (also ICSI) are billed from and reimbursed to the women. The actual distribution of costs within the family was not available.

The study plan was approved by the STAKES research ethics committee (18^th ^September 1998). For register linkages, the National Data Protection Authority was consulted and permissions from the registry keepers obtained.

## Results

During the mean 1.5 year study period, IVF women received on average 2.7 cycles (Table 1) resulting in at least one live birth for 40% of women (Table 2). The success rate per cycle was on average 15%.

**Table 1:** Numbers and rates (per 1000 female population) of women having received IVF and number of treatment cycles per treated women by women's background characteristics and treatment sector in the study period (mean 1.5 years).

	**Number**					**Population rate**					**Number of cycles per treated women**				
**Age**	20–29	30–34	35–39	40+	Total	20–29	30–34	35–39	40+	Total	20–29	30–34	35–39	40+	Total
**Socioeconomic position**															
Upper white-collar	367	856	727	368	2318	14	30	24	4	13	2.3	2.8	2.9	3.1	2.8
Lower white-collar	1072	1613	1256	510	4451	13	25	17	2	9	2.2	2.7	2.8	3.0	2.6
Blue-collar	460	479	372	173	1484	8	16	12	1	6	2.3	2.6	2.7	3.0	2.6
Others *	330	284	182	126	922	2	5	3	1	2	2.2	2.7	2.6	2.8	2.5
**Area of residence †**															
Urban	1439	2185	1780	800	6204	6	18	15	2	7	2.2	2.7	2.8	3.1	2.7
Semi-Urban	331	476	337	154	1298	8	17	11	1	6	2.1	2.8	2.8	3.0	2.6
Rural	454	566	415	215	1650	10	18	11	1	6	2.3	2.7	2.8	2.7	2.6
**Sector**															
Public	938	1242	873	175	3228	NA	NA	NA	NA	NA	1.9	2.2	2.4	2.2	2.2
Private	987	1585	1354	902	4828	NA	NA	NA	NA	NA	2.2	2.7	2.7	2.9	2.6
Both	304	405	310	100	1119	NA	NA	NA	NA	NA	3.1	4.4	4.6	5.3	4.2

**Total**	2229	3232	2537	1177	9175	7	18	14	2	7	2.2	2.7	2.8	3.0	2.7

* Entrepreneurs, students, pensioners, unemployed, and women with an unclassified or unknown position. † Excluding women with unknown area of residence (n = 23). NA = Not available

**Table 2:** Success of IVF treatment by women's background characteristics and treatment sector.

	Live births/cycle *					**Live births/treated women †**				
**Age**	20–29	30–34	35–39	40+	Total	20–29	30–34	35–39	40+	Total
**Socioeconomic position**										
Upper white-collar	0.25	0.18	0.13	0.06	0.15	0.55	0.49	0.38	0.20	0.42
Lower white-collar	0.23	0.17	0.14	0.05	0.16	0.49	0.46	0.37	0.15	0.40
Blue-collar	0.19	0.16	0.11	0.07	0.14	0.42	0.40	0.30	0.21	0.36
Others ^‡^	0.19	0.16	0.11	0.04	0.14	0.42	0.41	0.29	0.12	0.35
**Area of residence §**										
Urban	0.22	0.17	0.13	0.06	0.15	0.49	0.48	0.36	0.18	0.41
Semi-Urban	0.20	0.16	0.14	0.05	0.15	0.44	0.45	0.40	0.14	0.40
Rural	0.20	0.15	0.12	0.06	0.15	0.49	0.43	0.36	0.17	0.40
**Sector**										
Public	0.20	0.18	0.13	0.04	0.16	0.39	0.40	0.31	0.08	0.35
Private	0.26	0.20	0.15	0.06	0.17	0.56	0.50	0.39	0.19	0.42
Both	0.15	0.10	0.08	0.04	0.10	0.44	0.44	0.36	0.23	0.40

**Total**	0.22	0.17	0.13	0.06	0.15	0.47	0.45	0.36	0.17	0.40

* All live births and all cycles during the mean 1.5 years treatment period. † One live birth per woman during the mean 1.5 years treatment period. ‡ Entrepreneurs, students, pensioners, unemployed, and women with an unclassified or unknown position. § Excluding women with unknown area of recidence (n = 23).

The costs of different types of IVF cycles are given in Table 3. Of the total IVF costs, 36% came from drugs, 21% from interventions, and 42% from other direct costs (data not shown). During the mean 1.5 year follow-up, total costs per treated woman were EUR 6500, of which 40% were paid by women themselves (from private sources) and 60% from public sources (data not shown). In the private sector, the out-of-pocket payment was 50% and in the public sector 24% of total expenditures. The cost of a live birth was on average EUR 16 000 (Table 4). In the population-based calculation showing the distribution of IVF expenditures across the whole population of fertile-age women(aged 20–49), the expenditures during the study period of 1996 to 1998 were EUR 60 per woman (Table 5), of which EUR 24 were paid by women themselves and EUR 36 from public sources.

**Table 3:** Women's own and society costs of one IVF cycle by the type of treatment and by the treatment sector (euros).

	**Public sector**			**Private sector**			**All cycles**		
**IVF without embryo transfer**	Median	Mean	(Range)	Median	Mean	(Range)	Median	Mean	(Range)
Own	450	570	(130–5870)	1130	1350	(690–6170)	810	940	(130–6170)
Society	1290	1410	(980–6710)	920	1060	(380–5890)	1160	1250	(380–6710)
Total	1740	1980	(1110–12570)	2030	2410	(1070–11990)	1900	2180	(1070–12570)
**IVF with embryo transfer**									
Own	850	940	(170–4240)	1620	1750	(540–8860)	1340	1490	(170–8860)
Society	2960	3050	(2300–6330)	1790	1880	(520–7840)	2160	2260	(520–7840)
Total	3800	3990	(2470–10570)	3450	3640	(1060–16700)	3640	3750	(1060–16700)
**Frozen embryo transfer**									
Own	140	190	(110–2200)	380	540	(210–7060)	330	410	(110–7100)
Society	810	860	(800–2850)	450	580	(180–7310)	660	680	(180–7310)
Total	950	1050	(910–5040)	810	1130	(400–14370)	930	1100	(400–14370)

**Table 4:** Number of cycles needed to live birth, total expenditure and the proportion of public expenditure (%) per live birth by women's background characteristics

	**Number of cycles per live birth**					**Total expenditure, euros**					**Public expenditure, %**				
**Age**	20–29	30–34	35–39	40+	Total	20–29	30–34	35–39	40+	Total	20–29	30–34	35–39	40+	Total
**Socioeconomic position**															
Upper white-collar	4.1	5.6	7.5	15.8	6.6	12130	13200	17780	38440	16130	60	59	58	50	57
Lower white-collar	4.3	5.8	7.3	19.3	6.3	12260	13500	16930	43300	15300	63	62	61	52	61
Blue-collar	5.3	6.1	9.0	13.8	6.9	14300	14690	20500	31840	16910	65	63	62	53	62
Others *	5.2	6.3	8.9	23.8	7.1	14080	14600	20390	59310	17340	63	63	59	52	60
**Area of residence †**															
Urban	4.5	5.6	7.8	17.0	6.5	12400	13280	18120	41100	15830	62	61	59	51	59
Semi-Urban	4.8	6.2	6.8	22.2	6.6	14310	14310	16350	48700	16250	65	63	59	53	61
Rural	4.7	6.4	7.8	15.6	6.7	13400	14800	18030	33200	16100	64	64	62	53	62
**Sector**															
Public	5.0	5.6	7.6	27.3	6.1	13600	13370	17300	38770	14710	76	76	76	78	76
Private	3.9	5.3	6.9	15.8	6.2	10590	11840	16170	40520	14880	51	51	50	49	51
Both	6.9	10.1	12.8	23.0	10.5	20190	22580	26640	42830	23870	63	63	62	58	62

**Total**	4.7	6.0	7.8	17.4	6.7	12850	13660	17830	40660	15940	63	62	60	52	60

* Entrepreneurs, students, pensioners, unemployed, and women with an unclassified or unknown position. † Excluding women with unknown area of residence (n = 23).

**Table 5:** Total expenditure on IVF treatment * per female population by women's background characteristics (in euros).

	**Total expenditure, euros**				
**Age**	20–29	30–34	35–39	40–49	Total
**Socioeconomic position**					
Upper white-collar	100	200	170	50	110
Lower white-collar	80	160	110	20	80
Blue-collar	50	100	70	20	50
Others †	10	30	20	8	20
**Area of residence ‡**					
Urban	40	120	100	20	60
Semi-Urban	50	110	70	20	50
Rural	60	110	70	20	50

**Total**	40	120	90	20	60

* Excluding IVF women aged 50 years or more (n = 16). † Entrepreneurs, students, pensioners, unemployed, and women with an unclassified or unknown position. ‡ Excluding women with unknown area of residence (n = 23).

The use, success and expenditures of IVF were age-dependant. Numbers and rates of women having received IVF were highest among women aged 30–34 (Table 1), but the treatment was more intense for older women (number of cycles per woman) (Table 1). The success rate per cycle and per woman decreased by age (Table 2); the number of needed cycles for live birth increased by age (Table 4). The higher treatment intensity among older women did not compensate for the lowered success rate, and about 47% of women aged under 30 and only 17% of women aged 40 or older succeeded in achieving a live birth after the treatment period (mean 1.5 years) (Table 2).

The unit expenditure of IVF did not vary much between age-groups, but older women were paid more of the total expenditures from their own pockets; 37% among women under 30 years, 38% among women aged 30–34 years, 40% among women aged 35–39 years, and 48% among women aged 40–49 years. Total expenditures per live birth increased by age, being 3.6 times higher among older women compared to the younger women (Table 4). Expenditures per population were highest among women aged 30–34 and then decreased (Table 5), due to the lesser use of IVF among older women (Table 1).

The use, success and expenditures of IVF also varied somewhat by socioeconomic position. Women from the highest socioeconomic position received more cycles and on a population-based examination used IVF twice as much as the blue-collar women in every age-group (Table 1) as well as spent more of their own money for IVF treatment compared to blue-collar women regardless the age of the women (data not shown). About 25% of white-collar women aged under 30 succeeded in achieving a live-birth, but only 19% of blue-collar women after the mean 1.5 year treatment period succeeded (Table 2), while the number of needed cycles per live birth among blue-collar women was higher. Except for the oldest women, the total expenditure and the proportion of public expenditure were higher among blue-collar women than among upper-white collar women (Table 4). Due to the higher use of IVF, in every age group, the public expenditure was about two-fold for upper white-collar women compared to blue-collar women (Table 5).

Women treated in the private sector received more cycles than women in the public sector, and the women treated both in the public and in the private sector ('both sector users') received the most cycles (Table 1). Success was poorest among both sector users (Table 2) and their live births were the most costly (Table 4).

No remarkable regional differences were found according to the urbanity of the living area (Table 1, 2 and 4).

## Discussion

Among infertile women in the care system, older women with poorer success rates (i.e. increased need) received more treatments, and expenditures per live birth were much higher among them than among younger women. The expenditures per population were lower among older women, since fewer older women were treated. Because no data relating to the desire for a child by age were available, it is not certain whether all younger and older women wishing to have a baby were in the IVF care system. Women from a higher socioeconomic position had more often used IVF care, and total and societal costs per population were higher than among women from the lower socioeconomic position. Regional differences were not remarkable.

Are these results reliable? Our data was based on administrative registers, and we think that the results on treatment and success rates by socioeconomic characteristics are reliable. The group 'other' is a heterogeneous group that also includes entrepreneurs. Since we did not have any information on the size of their enterprise, we could not define a socioeconomic position for them. Due to the small proportion (2%), it is unlikely to bias our results based on socioeconomic position. However, grouping into private and public sectors and expenditures have to be interpreted with reservations due to the various estimations made. The cost estimates of interventions and examinations in private clinics were based on the prices in one clinic. But that clinic was one of the biggest and when we asked prices from several clinics, they recommended obtaining data from this clinic and to use it as the reference. Our basic data on expenditures of treatments and drugs were based on the situation in the late 1990s. We do not, however, consider this a drawback because all expenditures were inflated to correspond to 2005 prices, and we focused primarily on describing the differences in expenditures by women's age and socioeconomic position rather than the exact amount of euros. Furthermore, our cost estimation of a successful IVF cycle is in accordance with an earlier Finnish study [14] as well as earlier cost calculations by age that have included the same components as used in our study [10]. However, only some of the actual costs were taken into account, the costs excluded being non-routine radiological and laboratory tests and all indirect costs such as travel costs and sick leaves. Furthermore, the costs of complications as well as pregnancy and birth costs that are known to be higher among older women were not included. Had all costs been included, the total expenditures may have been higher for the older women and possibly also for rural women due to the longer distances travelling to care facilities. The impact on socioeconomic differences remains unclear.

Traditionally the discussion on unfairness in health care has focussed on socioeconomic position and gender and less on age. As expected, there were fewer older than younger women in the care system, because childbearing is usually started earlier than 35 years. The mean age of maternity is 30 years in Finland [16]. However, if older women sought IVF treatment, they received more cycles because of lower success, and the expenditure of a live-birth increased according to age due to the increased number of cycles and amount of expensive drugs in the cycles. Pregnancy and birth complications and poor foetal outcomes in general increase by age [17,18] suggesting that expenditures of pregnancy and child birth of older women would also increase by age. Thus, if IVF resources are scarce, it would be wise to concentrate on treating younger women. On the other hand, it can be argued that age should not be a reason to turn women away from IVF, because for older women, IVF may offer the last chance to become pregnant and have a child [19]. Should older women with greater need receive more resources than younger women? From a public health perspective treating older women with increasing costs and risks and decreasing success of IVF is not wise. Furthermore, if the older women's desire for children increases i.e. care seeking increases, which has already been reported in the United Kingdom [20], the problem of resource allocation will grow in the future. Both the scarce health care resources and the increasing need for prioritisation speak for focusing treatments on women in their normal childbearing age.

Live births were most costly among 'both sector users' in our study. Their success per cycle was the poorest, but due to many attempts they succeeded as well on average as all women taken in total in this study; 40% of them received a child. Some of them may have been women who went to a private clinic while being on the waiting list (i.e. waiting for access to care) for the public sector [21]. Some may have been women who had poor prognosis and after unsuccessful treatments in one clinic sought treatments in other clinics. From 1996 to 1998 in Finland no waiting lists existed in the private sector but in the public sector couples had to wait between one and two years. In 2005 the situation had changed due to the new time-frames for receiving care stipulated by the Ministry of Social Affairs and Health [22]. The longest waiting period is stipulated to be 6 months in the public sector. However, an age-limit of 40 years is still in force in the public sector.

In Finland, over 60% of IVF treatments are provided in the private sector and women from the highest socioeconomic position are overrepresented in the private sector [7]. In spite of the reimbursement of private services, the private expenditure remains higher i.e. more of women's own financial resources are needed for treatments in the private sector compared to the public sector. The use of private services is likely to create unfairness also in Finland. However, the unfairness is smaller than in countries where IVF is not covered by health insurance [23,24]. In the United Kingdom where 80% of IVF is given in the private sector, authorities have warned that IVF is becoming more commercial and people with insufficient income are in danger of remaining without treatments [25]. According to the review by Dawson *et al.*[26], the most common reasons for inability to obtain IVF treatment have been financial.

It can be asked what priority should be given to infertility treatments compared to other treatments in health care. As in other countries [4,27,28], prioritizing has been indirectly discussed in Finland: Should infertility be considered a disease or not, should treatments be given only for medical reasons (diagnosed infertility) or also for social reasons, and who should have the right to treatments or eligibility? Prioritization has not, however, been discussed explicitly, even though IVF is clearly prioritized by women's age.

For some infertile couples the adoption of a child is an alternative to infertility treatment. However, adoption is costly for couples, too. In Finland, only foreign adoptions are available. It can be argued that, if a part of the costs of infertility treatments are covered from public funds, the adoption costs should be covered, too. In the late 1990s Finnish couples had to pay all the expenditures of adoption themselves, but since 2003 parents adopting a child from abroad can claim an adoption grant from the Social Insurance Institution to offset some of the adoption cost. The adoption grant is a tax-free, one-time payment. Its amount depends on the child's country and varies between EUR 1900 to EUR 4500. According to present study, the society covers about 60% of IVF expenditures i.e. EUR 9600 per IVF live birth, which is about 2- to 5- fold compared to the coverage of adoption.

Besides financial factors other factors also related to socioeconomic position may be important in seeking or using infertility treatments. They include recognition of fertility problems, attitudes towards health and medical treatment, social support, health status, and prior contacts and experiences with health care [6,29].

In our study expenditures per live birth were somewhat higher among women from the lower socioeconomic position, but due to far more frequent use of IVF services, the expenditures per population were greater among women from higher socioeconomic position. Does this indicate unfairness in the care provision? If fewer blue-collar women were not as willing to use or did not need IVF services as much as white-collar women, this differentiated allocation cannot be considered as inequality. But if services are organized such that women do not have the same possibility to use them, it is unfair [30]. In this study it was not possible to examine the reasons for the use or non-use of IVF. Neither was it possible to study whether the treatments or the quality of care varied by different socioeconomic positions. In France, despite equal use of IVF, a deeper analysis showed that women from lower positions faced greater risks and lower benefits [6]. However, based on the fact that the most of IVF is given in the private sector and that highly educated people are used to going to the private sector compared to people with lower education we think that the latter is more likely in Finland. Poorer success (live-births per treated woman and higher number of cycles per live birth) among blue-collar women in our study may be related to more serious infertility; infertility-related risk factors like smoking and obesity are more common among blue collar women [31].

Access to IVF services can vary between geographical areas even in countries where IVF is publicly funded [32]. In Finland we did not find remarkable regional differences in the use of IVF [7] and likewise in the current study, there were no differences found in the resource allocation.

It is difficult to determine an optimal and equal age to stop the resource allocation for IVF. Treatment of selected women aged 40–43 has been found to be quite successful [10,19] and natural pregnancies also occur for women in their forties. However, a given age-limit could be a signal that encourages women to attempt to give birth during the normal fertile age. Social and family policy should promote circumstances that are suitable for childbearing at a young age. As Chavkin [33] and Stillman [34] have pointed out that this is a big challenge especially for countries that lack policies supporting parents who both work.

## Conclusion

This study shows that the resources used for IVF varied by women's age and socioeconomic position. IVF-children of older women involve more expense due to the lower success rates of IVF. Women from higher socioeconomic positions used more IVF resources than other women. Women and men need information on declining fertility by age as well as the complexity of IVF. Causes of unequal use of IVF services should be studied further, and resource allocation for high technology should be made transparent.

## List of abbreviations

ICSI = Intra cytoplasmic sperm injection

IVF = In vitro fertilization

## Competing interests

The authors declare that they have no competing interest regarding this nationwide register-based cohort study.

## Authors' contributions

RK collected and analysed the data and drafted the manuscript. MG contributed to conception and design of the study and interpretation of the data. TS participated in the design and analysis of the study. EH made substantial contributions to conception and design of the data as well as interpretation of the data and helped in drafting the manuscript through critical revision. All authors read and approved the final manuscript,

## Pre-publication history

The pre-publication history for this paper can be accessed here:



## Supplementary Material

Additional file 1 Type of IVF cycle, expenditures included in cost calculations in each type of cycle, as well as type and data source of expenditures by care sector. Click here for file

## References

[B1] Graigner DA, Tjaden BL, Goldman MB, Hatch MC (2000). Assisted Reproductive Technologies. Women and Health.

[B2] Evers JLH (2002). Female subfertility. Lancet.

[B3] Notkola I-L (1995). Uutta tietoa hedelmättömyyden yleisyydestä. [New information about infertility prevalence.] (In Finnish). Suomen Lääkärilehti.

[B4] Neumann PJ (1997). Should health insurance cover IVF? Issues and options. J Health Polit Policy Law.

[B5] Stephen EH, Chandra A (2000). Use of infertility services in the United States: 1995. Fam Plann Perspect.

[B6] Tain L (2003). Health inequality and users' risk-taking: a longitudinal analysis in a French reproductive technology centre. Soc Sci Med.

[B7] Klemetti R, Gissler M, Hemminki E (2004). Equity in the use of IVF in Finland in the late 1990s. Scan J Public Health.

[B8] STAKES Finnish IVF-statistics 1992–2001 – Data Supplier Feedback 2002.

[B9] Svensson P-G, Stephenson P, Stephenson P, Wagner MG (1993). Equity and resource distribution in infertility care. Tough choices: In vitro fertilization and the reproductive technologies.

[B10] Broekmans FJ, Klinkert ER (2004). Female Age in ART: When to stop?. Gynecol Obstet Invest.

[B11] Hemminki E, Klemetti R, Rintapaavola M, Martikainen J (2003). Identifying Exposures from Drug Reimbursement Files: A Study on Unintended Effects of In-Vitro-Fertilization in Finland. Med Inform Internet Med.

[B12] Statistics Finland. http://tilastokeskus.fi/tk/tt/luokitukset/lk_en/sosioekon_asema_index.html.

[B13] Klemetti R, Sevón T, Gissler M, Hemminki E (2005). Complications of IVF and ovulation induction. Hum Reprod.

[B14] Koivurova S (2005). In vitro fertilization in Northern Finland 1990–1995. Prenatal and early childhood outcome until three years of age. PhD Thesis, University of Oulu.

[B15] Klemetti R, Sevón T, Gissler M, Hemminki E (2006). Health of Children Born as a Result of In Vitro Fertilization. Pediatrics.

[B16] STAKES (2006). The Finnish Medical Birth Register.

[B17] Nybo Andersen AM, Wohlfart J, Christens P, Olsen J, Melbye M (2000). Maternal age and fetal loss: population based register linkage study. BMJ.

[B18] Salihu HM, Shumpert MN, Slay M, Kirby RS, Aleksander GR (2003). Childbearing beyond Maternal Age 50 and Fetal Outcomes in the United States. Obstet Gynecol.

[B19] Klipstein S, Regan M, Ryley DA, Goldman MB, Alper MM, Reindollar RH (2005). One last chance for pregnancy: a review of 2,705 in vitro fertilization cycles initiated in women aged 40 years and above. Fertil Steril.

[B20] (2007). A long term analysis of the HFEA Register data (1991–2006). Human Fertilisation & Embryology Authority.

[B21] Malin Silverio M, Hemminki E (1996). Practice of in-vitro fertilization: a case study from Finland. Soc Sci Med.

[B22] The Ministry of Social Affairs and Health (2005). Yhtenäiset kiireettömän hoidon perusteet. *Sosiaali- ja terveysministeriön oppaita 2005:5 *[Uniform Access to non-emergency treatment, *Handbooks of the Ministry of Social Affairs and Health 2005:5*].

[B23] Filetto JN, Makuch M (2005). Long-term follow-up of women and men after unsuccessful IVF. Reprod BioMed Online.

[B24] Jain T, Hornstein MD (2005). Disparities in access to infertility services in a state with mandated insurance coverage. Fertil Steril.

[B25] Cole A (2006). Assisted conception business should be better regulated. BMJ.

[B26] Whitehead M (1990). The concepts and principles of equity and health. World Health Organization, Regional Office for Europe.

[B27] Nisker JA (1996). Rachel's ladders or how societal situation determines reproductive therapy. Hum Reprod.

[B28] Daniels K, Taylor K (1993). Formulating selection policies for assisted reproduction. Soc Sci Med.

[B29] Dawson AA, Diedrich K, Felderbaum RE (2005). Why do couples refuse or discontinue ART?. Arch Gynecol Obstet.

[B30] White L, McQuillan J, Greil AL (2006). Explaining disparities in treatment seeking: the case of infertility. Fertil Steril.

[B31] Helakorpi S, Patja K, Prättälä R, Uutela A (2001). Health behavior and health among Finnish adult population, spring 2001. Publications of the National Public Health Institute B16/2001.

[B32] Blank RH (1997). Assisted reproduction and reproductive rights: the case of in vitro fertilization. Politics Life Sci.

[B33] Chavkin W (2007). Older Age, Infertility, and Multiple Pregnancies. JAMA.

[B34] Stillman RJ (2007). Older Age, Infertility, and Multiple Pregnancies. JAMA.

